# A roadmap for maximizing the use and effectiveness of recovery housing for individuals prescribed medications for opiate use disorders

**DOI:** 10.3389/fpubh.2025.1533082

**Published:** 2025-07-04

**Authors:** Amy A. Mericle, Carmen L. Masson, Sarah E. Zemore, Meenakshi S. Subbaraman, Dustin Khebzou, Diane Schmidt, Kwinoja Kapiteni, Leonard A. Jason

**Affiliations:** ^1^Alcohol Research Group at the Public Health Institute, Emeryville, CA, United States; ^2^Weill Institute for Neurosciences, University of California, San Francisco, San Francisco, CA, United States; ^3^Behavioral Health and Recovery Studies Program at the Public Health Institute, Oakland, CA, United States; ^4^Center for Community Research at DePaul University, Chicago, IL, United States

**Keywords:** addiction, recovery, opioid use disorder, medications for opioid use disorder, recovery housing, recovery residences

## Abstract

**Introduction:**

Safe and stable housing is increasingly recognized as critical to recovery from alcohol and drug use disorders, but research on the outcomes of residents in recovery from opioid use disorder (OUD), particularly those prescribed medications for opioid use disorder (MOUD), is limited.

**Methods:**

This article presents results from an informal survey (*N* = 15) and discussion with experts in the recovery housing and OUD treatment fields serving as Advisory Board members on the Infrastructure for Studying Treatment and Addiction Recovery Residences (I-STARR) project regarding priorities for research and training on recovery housing for individuals prescribed MOUD. Drawing on the results, we provide a roadmap to establish an evidence base on recovery housing for those prescribed MOUD.

**Results:**

Three of the highest-ranked research topics identified were: (1) Assessment of recovery housing outcomes of people prescribed MOUD and factors that may influence outcomes; (2) Examination of factors associated with MOUD adherence among recovery housing residents; and (3) Strategies to increase linkage between MOUD prescribers/treatment providers and recovery residence operators. Additional topics emerged during discussion, most prominently the examination of barriers to, and facilitators of, accessing recovery housing among people prescribed MOUD. The highest-rated training topic for researchers and recovery housing operators was challenges faced by recovery housing operators.

**Conclusion:**

Research is urgently needed to establish an evidence base on recovery housing for those prescribed MOUD, and both researchers and operators in the field would benefit from training to ensure that potential challenges to moving research forward on this topic are addressed.

## 1 Introduction

The ongoing opioid crisis continues to represent an urgent public health priority (1, 2). Opioids are potent modulators of many physiological and psychological processes, making them highly addictive. Specifically, repeated or chronic use of opioids modifies neuronal circuitry, leading to tolerance, dependence, and withdrawal and places one at risk for opioid use disorder (OUD) (3). Individuals with OUD have increased medical comorbidity and impairments in a wide range of domains (4); moreover, opioid use can be deadly. In 2022, 107,941 drug overdose deaths occurred, the majority of which involved an opioid, resulting in an age-adjusted rate of 32.6 deaths per 100,000 standard population (5). In addition to the human toll, the economic burden of OUD and fatal opioid overdose in 2017 was estimated to be $1.02 trillion, with the majority of costs resulting from reduced quality of life and the value of life lost due to fatal opioid overdose (6).

Treatment of OUD is a key component in the response to this crisis (1). Medications for OUD (MOUD), which include methadone, buprenorphine, and extended-release naltrexone, significantly improve opioid outcomes (7). However, the effectiveness of MOUD is limited by problems at all levels of the substance use continuum of care, including entry into MOUD treatment, receipt of these medications, and retention in treatment (8). MOUD alone also may be insufficient to assure long-term reductions in use and return to functioning. Recognizing the chronic nature of addiction and recovery as both an organizing construct and a goal for substance use treatment, a variety of different recovery support services have emerged to augment and extend treatment services (9). Unlike recovery support received within the context of 12-step groups (e.g., AA, NA), these services are delivered by trained peers or other professionals. These services include various peer-based recovery support services (e.g., recovery coaches, peer navigators), clinical continuing care, recovery community centers, activity-based recovery communities, educationally based recovery supports (e.g., recovery high schools and collegiate recovery communities), as well as *recovery housing*.

### 1.1 Recovery housing for those with OUD

Recovery housing is defined by the Substance Abuse and Mental Health Services Administration (SAMHSA) as safe, healthy, family-like substance-free living environments that support individuals in recovery from addiction (10). Although residences can vary widely in structure and nature of services provided (ranging from self-governed residences, like Oxford Houses to treatment settings, like therapeutic communities [TCs]), peer support and resident responsibilities within the household community are hallmarks of recovery housing. Further, “home,” having a safe and stable place to live, is integral to recovery (11), and recovery housing is an increasingly common way for those in recovery to experience the benefits of it. A national survey of individuals who had resolved a problem with alcohol or drugs found that 9% had accessed recovery housing; notably, only 28% reported accessing any formal treatment (12).

Mirroring national trends, OUD is growing in prevalence among those in recovery housing. One study found that 19% of residents recruited at intake into sober living houses in Los Angeles met diagnostic criteria for opioid dependence (13). For those in the early phases of OUD recovery, recovery housing could provide a safe living environment and structure for residents (14–19), potentially minimizing exposure to conditioned cues and other triggers for relapse and helping with medication adherence. It could also lay the foundation for long-term gains by helping residents develop invaluable recovery skills, such as managing withdrawal symptoms and cravings and connecting residents with new peer communities (20–25), which is critical given that opioid-related brain changes can take months or years to resolve. Recovery housing is based on the principles of social model recovery, which emphasize creating a home-like and recovery-supportive environment, underscoring the importance of abstinence-based social networks, experiential knowledge, and non-hierarchical relationships between staff and residents (26, 27). Social support is critical to recovery (28). Social support offered within recovery housing may be particularly valuable for individuals prescribed MOUD who often encounter stigma in more traditional mutual-help settings (29, 30). According to the social identity model of recovery (SIMOR), these kinds of settings can facilitate recovery through socially negotiated identity transformational processes, helping one shift from being defined by membership of a group whose norms and values revolve around substance used to being defined by membership of a group whose norms and values encourage recovery (31). Recognizing its potential role in stemming the opioid crisis, several of the SAMHSA guidelines for recovery housing focus specifically on MOUD. These include promoting the use of evidence-based treatments in recovery housing, like MOUD, and developing and implementing medication policies to ensure resident safety.

### 1.2 Research on MOUD and recovery housing

Although research on recovery housing is more mature than for some other types of recovery support services, a number of key gaps remain. Over three decades of research involving several large studies has led to TCs becoming recognized by the National Institute on Drug Abuse as an effective treatment approach (32). The evidence base for other types of recovery residences is strong as well. A national outcome study and two randomized trials have consistently demonstrated decreased substance use, decreased involvement in the criminal justice system, and higher incomes among residents recruited from Oxford Houses (33–36). Studies of other types of recovery residences are fewer in number but also promising. Three separate longitudinal studies of residents living in sober living houses in California have also found improvements in substance use, arrest rates, and other problem severity (13, 37, 38). Studies of recovery housing with more services and structure and in different parts of the country are even fewer in number but have also found that recovery housing is associated with improved substance use treatment outcomes (39) and increased recovery capital (40, 41). Yet despite this research, the evidence base has been criticized for its lack of geographic diversity and representation of varying types of recovery residences (42) as well as for how few studies have been conducted (43) and the dearth of researchers conducting this work (44).

Additionally, few studies have examined recovery housing for persons with OUD, and research examining the outcomes of residents prescribed MOUD is even more notably lacking. Tuten et al. (45) found that abstinence following medication-assisted detoxification in opioid-dependent adults improved among those receiving recovery housing versus usual care. The addition of intensive “reinforcement-based treatment” further improved treatment outcomes, in part by promoting longer recovery house stays. In another study, these investigators found that opioid-dependent individuals receiving recovery housing, regardless of how it was paid for, had superior abstinence outcomes compared to those receiving reinforcement-based treatment (46). In sum, this research points to recovery housing being beneficial to those with OUD, but little information was provided on the recovery housing received, limiting conclusions about what is helpful. Moreover, these studies focused on receipt of recovery housing without examination of potential barriers to entry into recovery housing among individuals with OUD, and individuals prescribed opioid agonist medications (i.e., methadone or buprenorphine) were excluded.

The research that has focused on the topic of MOUD and recovery housing has highlighted operator-level barriers, both logistical (e.g., staffing and providing needed safeguards around medications) and attitudinal (e.g., negative beliefs and biases), to supporting residents on MOUD (47–49), pointing to an urgent need to train and support recovery housing operators on best practices and around addressing the needs of individuals taking MOUD. Yet research also suggests that house composition, namely having residents in the house being treated with MOUD is associated with more favorable resident attitudes toward MOUD (50); and for those being treated with MOUD, living among other residents on MOUD has been found to lessen the effects of psychiatric severity on stress (51) and enhance the buffering effects of social support on stress (52). Moreover, qualitative work examining the experiences of residents in recovery housing developed specifically to serve people taking MOUD highlights how pivotal such housing can be for those taking MOUD (53, 54). Taken together, these findings underscore the need to reduce barriers within recovery housing for those being treated with MOUD (55) and to study the effectiveness of recovery housing for this population.

### 1.3 Enhancing MOUD and recovery housing research infrastructure: the I-STARR project

Recovery housing could play a key role in the nation’s response to the ongoing opioid crisis, but barriers to recovery housing for those being treated with MOUD abound, and we lack research on its effectiveness to support this population. The Infrastructure for Studying Treatment and Addiction Recovery Residences (I-STARR) project was developed to increase the capacity of recovery housing equipped to support residents prescribed MOUD and to enhance the skills of those conducting research on it by providing web-based trainings to operators (from recovery residence providers to house managers) and researchers. In connecting recovery housing operators and researchers in a virtual learning community, and by funding pilot studies with the dual purposes of beginning to address key questions about recovery housing for individuals taking MOUD and developing greater depth and diversity among researchers conducting studies on this topic, the I-STARR project seeks to seed research that will lead to the identification of evidence-based practices around MOUD in recovery housing. I-STARR activities are guided by an advisory board, which was convened at the outset of the project to prioritize topics for research as well as training areas for researchers and recovery housing operators to support this work. In this brief research report, we present the results of an informal survey of and discussion with the I-STARR Advisory Board members, whose input was used to establish a roadmap to prioritize research on the topic of recovery housing for those prescribed MOUD and training needed to conduct it.

## 2 Methods

Our approach for setting research and training priorities was guided by the Child Health and Nutrition Research Initiative (CHNRI) (56), which outlines a systematic method for setting priorities in health research, and principles set forth by the James Lind Alliance (57), which promotes the use of priority setting partnerships where diverse stakeholders (e.g., patients, carers and health professionals) collaborate to identify priority topics for new research. The blending of these approaches resulted in a six-stage process (see Table 1), incorporating both primary and secondary research activities as well as quantitative and qualitative methods. I-STARR project activities are approved and monitored by the Public Health Institute Institutional Review Board. The Advisory Board, which regularly meets at least twice yearly, was convened in the spring of 2023 to prioritize research and training activities.

**Table 1:** I-STARR priority setting process and activities.

Stage	Activities
1. Pre-meeting activities	(a) Identification of diverse stakeholders, the I-STARR Advisory Board, to shape priority setting and investigator driven “evidence uncertainty” identification; and (b) systematic listing of proposed research and training topics from review of the extant literature.
2. Initial scoring of and stakeholder feedback on research and training topics	(a) Rating (on a scale ranging from 1 (Low Priority) to 5 (High Priority) pre-identified topics; and (b) solicitation of responses to prompts for other topics.
3. Review and discussion of ratings with advisory board members	(a) Review of average ratings (sorted from high to low) and suggestions of other topics with the full Advisory Board; (b) breakout rooms to refine training topics for researchers and operators/providers; and (c) further discussion with the full Advisory Board to summarize discussion in the breakout groups and invite members to provide further comments.
4. Post-meeting activities	(a) Review and synthesis of Advisory Board member discussion; and (b) Investigator identification of six research priorities for I-STARR pilot studies
5. Publication and dissemination	(a) Research priorities for pilot studies and training topics identified on the project website; and (b) Pilot study priorities and training topics listed in project newsletters and email outreach notifications
6. Implementation and revision	(a) Priorities used to guide the selection of pilot studies for funding; and (b) Priorities and topics are revisited and augmented during quarterly Advisory Board meetings.

### 2.1 Participants

At the time of the spring meeting, the I-STARR Advisory Board was comprised of a diverse group of 18 members from across the country (see Table 2). Then and now, Advisory Board members consist of recovery housing and health services researchers with expertise in conducting research in treatment, criminal justice, and community-based settings—all settings in which there are individuals prescribed MOUD who could potentially benefit from recovery housing. The Advisory Board also includes representatives of national organizations whose membership represents both treatment and recovery housing providers. Finally, members also include treatment providers (including two medical doctors) and operators of recovery housing who could address potential barriers to supporting individuals on MOUD in recovery housing as well as payors for recovery housing at the local level. To further ensure diversity among the Advisory Board members, we purposely included those who could offer differing perspectives on MOUD, individuals with quantitative as well as *qualitative* and community-participatory research expertise, and members with lived experience in recovery and with recovery housing.

**Table 2:** Advisory board member characteristics (*N* = 18).

	n	%
Region represented		
National	5	28%
Northeast	3	17%
South	2	11%
Midwest	4	22%
West	4	22%
Profession		
Researcher/Educator	8	44%
Provider/Payor	7	39%
Advocate	3	17%
Area of expertise		
OUD/MOUD/Addiction	4	22%
Recovery housing/Recovery support	10	56%
Substance use/Community-based treatment	4	22%
Female	6	33%
Racial/ethnic minority	6	33%
Persons in long-term recovery	4	22%

Advisory Board members bring a wealth of professional experience and expertise to the role (i.e., clinician researchers who have conducted research in a variety of areas), but some were invited to serve because of specific experience and expertise highlighted in the table. Of the researchers/educators represented, half have experience conducting qualitative research, with two having specific expertise in qualitative methods or community-based participatory research methods.

### 2.2 Data collection, measures, and analytic procedures

A week before the scheduled Advisory Board meeting, members received an email reminder about the meeting. The email also contained the meeting agenda, a list of topics with descriptions of potential webinars in the researcher and the operator training series, and a link to an anonymous survey to collect input regarding research and training topics that should be addressed by the project.

The first section of the survey (see Supplementary Appendix A) asked all Advisory Board members to rate eight topics, identified by members of the I-STARR team as gaps in the literature, on a five-point scale according to their perceived priority for research funding (1 = lowest and 5 = highest). Topics included measurement development, barriers/facilitators to accessing recovery housing among those prescribed MOUD, and outcomes of people living in recovery housing who take MOUD, to name a few. This section also asked members to suggest additional research topics relevant to MOUD in recovery housing settings. The next section asked Advisory Board members to rate, using the same scale, topics under consideration by the I-STARR team for the researcher training series. This included nine researcher-specific topics [methods and topics identified as being particularly challenging for recovery housing researchers—see (44)], and three topics germane to researchers and recovery housing operators (referred to as “all-audience” webinars). This section also included an open-ended question querying other topics and implemented skip logic to reduce respondent burden for those who identified as recovery housing operators. The final section asked Advisory Board members to rate topics currently under consideration for the operator training series, comprised of nine recovery housing operator-specific topics (general recovery housing best practices and those specific to MOUD identified by national recovery housing experts) and the three “all-audience” webinars. Like the prior section, it also included an open-ended question querying other topics and implemented skip logic for those who identified as recovery housing researchers.

The survey was completed in advance of the meeting by 15 members of the Advisory Board. These findings (averaged and sorted topic scores as well as lists of other suggested topics) were shared at the Advisory Board meeting, which was attended by all but one member (*N* = 17), as a way to ensure that those who were unable to complete the survey in advance could also register their priorities and to further stimulate discussion on both the research and training topics, particularly those that were not queried in the survey. The research priorities were discussed by all Advisory Board members in attendance. The meeting attendees were then split into breakout groups, with each of the mPIs leading either the discussion of the researcher training or the discussion of the provider training. Individuals who were not primarily a researcher or a provider (e.g., those who functioned largely as advocates) were randomly assigned to one of the groups. The two groups then came together to summarize each of the breakout discussions, with the mPIs summarizing the input from breakout group members. This time was also used to formulate final thoughts on both the research and training priorities. The meeting was recorded and impressions of it were further discussed by team members, particularly regarding the topic of research priorities.

## 3 Results

### 3.1 Research priorities

Table 3 lists the research topics queried in the survey, sorted by average priority score. Of those topics queried, the assessment of outcomes of people living in recovery housing who receive medications for opioid use disorder (MOUD) and factors that may influence outcomes ranked highest, and the study of social networks among residents in recovery housing receiving MOUD ranked lowest. That said, the average score for each topic was above 3 and only the top-rated topic was statistically different from the average priority rating across all topics. Other topics written in by Advisory Board members fell into three general categories pertaining to: types of effectiveness studies (e.g., effectiveness studies comparing different types of MOUDs, tapering strategies, and levels of support provided to those on MOUD); studies examining house-level factors (e.g., process research investigating how recovery housing is addressing residents’ needs, MOUD training strategies among recovery housing operators and providers, and examining state and county support for MOUD-capable recovery housing); and studies focusing on resident characteristics and experiences (e.g., disparities in accessing recovery housing among those taking MOUD, examining the mental health needs of among residents receiving MOUD, and MOUD access barriers among those in recovery housing).

**Table 3:** Research priorities sorted by average score (*N* = 15).

Research topic	Avg Score (1–5)	SD
Assessment of outcomes of people living in recovery housing who receive medications for opioid use disorder (MOUD) and factors that may influence outcomes	4.6	0.5***
Examining factors associated with MOUD adherence among recovery housing residents	4.1	1.1
Strategies to increase linkage between MOUD prescribers/treatment providers and recovery residence operators	4.0	1.0
Identification of individual, provider, and system-level barriers and facilitators to accessing recovery housing among those receiving MOUD	3.6	1.2
Identifying strategies to overcome recovery housing operator-level barriers to supporting residents receiving MOUD	3.6	1.1
Assessment of recovery housing residents’ perceptions of their MOUD treatment experience	3.5	1.1
The development of measures to assess the capability of recovery residences to oversee self-administration of and adherence of medications for opioid use disorder (MOUD).	3.5	1.1
The study of social networks among residents in recovery housing receiving MOUD	3.3	1.5

The average score across topics was 3.8. One-sample Student’s *t*-tests were performed to determine whether average topic scores varied significantly from the average score across items. *p < 0.05; **p < 0.01; ***p < 0.001.

**Figure 1:**
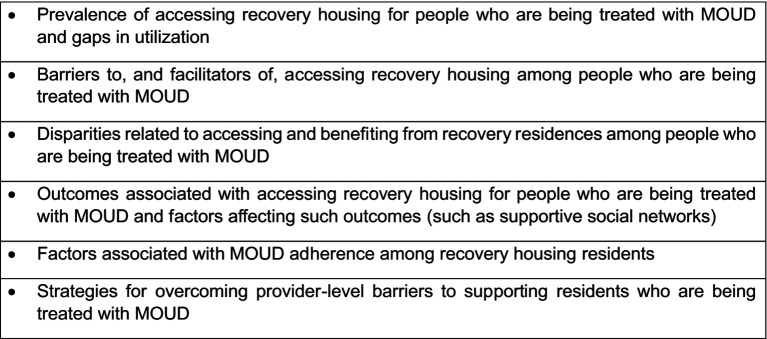
Pilot study research topics (*N* = 17).

During discussion with the Advisory Board at the meeting, the members reaffirmed the need for effectiveness and comparative effectiveness research on this topic but also noted that, if the project was focused on supporting pilot study research leading to those types of studies, other more basic research topics should also be considered. This more descriptive work could focus on areas such as the prevalence of accessing recovery housing for people who are being treated with MOUD and gaps in utilization. These sorts of descriptive studies would naturally lend themselves to studies of barriers to and facilitators of accessing recovery housing among people who are being treated with MOUD, including disparities related to accessing and benefiting from recovery residences among people who are being treated with MOUD. Smaller scale studies could still examine outcomes associated with accessing recovery housing for people who are being treated with MOUD as well as factors affecting such outcomes (such as supportive social networks). This work could also fit with studies examining factors associated with MOUD adherence among recovery housing residents as well as strategies for overcoming provider-level barriers to supporting residents who are being treated with MOUD. Topics crystalized in this process are listed in Figure 1, and they have been posted on the I-STARR website and used in funding announcements soliciting pilot study proposals.

### 3.2 Researcher and operator training topics

Table 4 lists the training topics that were queried separately by training track (researcher training and recovery housing operator training) and sorted by average priority score. The highest-rated training topic for recovery housing researchers was the challenges faced by recovery housing operators, and the lowest was statistical methods to strengthen causal inference. The average score for each topic was 3 or above, and three topics were statistically different from the average priority across all topics: the highest-ranked topic and two of the lowest-ranked topics. Discussion in the breakout group reaffirmed the importance of all topics, particularly the highest-ranked topic, underscoring the importance of researchers gaining an understanding of recovery housing before potentially questioning or investigating what may or may not be already in place. However, the group also reinforced the importance of training in qualitative techniques given the state of the science on this topic and that those conducting pilot study research would be doing more descriptive and formative work.

**Table 4:** Researcher (*N* = 14) and operator/provider (*N* = 9) training priorities sorted by average score.

Training topic	Avg Score (1–5)	SD
Researcher training		
Challenges faced by recovery housing operators/providers	4.1	0.7*
Recruitment and retention of marginally housed and legal-system-involved study participants	4.1	0.9
Basic information on recovery housing, like different types and services provided, the history and evidence base, current research gaps, and obstacles to conducting research^Ŧ^	4.0	0.8
Overcoming potential challenges to moving research forward on MOUD in recovery housing^Ŧ^	3.9	3.9
Sampling and collaborating with recovery housing operators/providers	3.8	1.1
Principles and components of community-based participatory research	3.6	0.9
Experimental and quasi-experimental recovery housing research designs	3.6	0.9
Multilevel modeling and studying “contextual” factors affecting residents	3.6	1.1
Recovery housing mechanisms of action and measurement of key constructs	3.5	0.9
Treatments and medication for opioid use disorder (MOUD) and pharmacotherapies for (OUD)^Ŧ^	3.4	1.0
Strengths and weakness of mixed-methods designs	3.0	0.7**
Statistical methods to strengthen causal inference	3.0	1.0*
Operator/provider training		
Overcoming potential tensions between prescribers and residence managers/operators	4.4	0.7*
Resident policies regarding admission and discharge, resident rights and responsibilities, substance use screening, confidentiality and releases of information, and handling grievances	4.0	1.1
Screening applicants prescribed MOUD and other medications of concern	4.0	0.9
Law/ethics/fiscal responsibility	3.9	0.6
Psychosocial interventions and medications for opioid use disorder (MOUD)^Ŧ^	3.8	0.8
Organizational-level challenges that need to be addressed when accepting residents on MOUD	3.8	0.8
Overcoming potential challenges to moving research forward on MOUD in recovery housing^Ŧ^	3.8	0.8
Basic information on recovery housing, like different types and services provided, the history and evidence base, current research gaps, and obstacles to conducting research^Ŧ^	3.7	0.9
MOUD diversion and risk management	3.7	1.0
Components of support provided within recovery housing and how this can be delivered	3.6	1.0
Fundamentals of recovery housing management and operations	3.2	1.2
Basic principles of research and participating in a research study	3.1	0.9

Researcher training topics were prioritized by eight Advisory Board members who identified as researchers and six members who identified as something else. Provider training topics were prioritized by three service (recovery housing or substance use treatment) providers and six members who identified as something else. ^Ŧ^All-audience webinar intended for recovery housing operators/providers as well as researchers (and any other key stakeholders). The average score for the Researcher Training topics was 3.6; for Operator/Provider Training topics, it was 3.7. One-sample Student’s *t*-tests were performed to determine whether average topic scores varied significantly from the average score across items. *P < 0.05; **P < 0.01; ***P < 0.001.

With respect to the training topics for recovery housing operators, the highest-ranked topic was overcoming potential tensions between prescribers and residence managers/operators, and the lowest-ranked topic was basic principles of research and participating in a research study. The average score for each topic was above 3, and only one topic was statistically different from the average priority across all topics: the highest-ranked topic. Discussion in the recovery housing operator training breakout group reaffirmed the importance of all topics, but those in this breakout group also identified new topics for this audience. Multiple members in this breakout group highlighted the importance of recovery housing operators forming partnerships and linkages to better support residents. Despite the lower rating for basic principles of research, members also noted the importance of helping operators conceptualize outcomes and better leverage data collected in the process of service delivery to facilitate outcome evaluation.

## 4 Discussion

The I-STARR project was developed to increase the capacity of recovery housing equipped to support residents taking MOUD and enhance the skills of those conducting research on it. The ongoing opioid crisis underscores the importance of research on this topic, and limited research resources should be put where they are most needed. With the input of the I-STARR Advisory Board, the project developed a roadmap for research on this topic, identifying research priorities as well as key topics that are critical to address with researchers and with recovery housing operators to conduct this research.

### 4.1 Research priorities

While the Advisory Board members reaffirmed the importance of effectiveness and comparative effectiveness studies, they also provided clear priorities with respect to the types of research that would be instrumental in moving the field forward to facilitate this research. These priorities have been used to guide the application and selection process for funding I-STARR pilot studies. To date, the project has supported four pilot studies: (1) a mixed methods study developing measures of attitudinal and capacity barriers in supporting residents taking MOUD in recovery housing; (2) a qualitative study examining the critical elements of recovery residences serving those taking MOUD who are involved in the criminal legal system; (3) a study examining MOUD utilization among recovery home residents with OUD in Philadelphia; and (4) a mixed methods study examining the potential of a retail mobile pharmacy to bridge access barriers among patients taking MOUD with recovery housing. In addition to filling important gaps, we fully expect these projects to lead to the planning of larger studies, facilitating much-needed effectiveness research.

### 4.2 Training priorities

Trainings identified by the team were all generally considered important to the Advisory Board members, with topics pertaining to recovery housing operations (either challenges faced by recovery housing operators or overcoming potential tensions between prescribers and residence managers) being rated most highly for both researcher and operator audiences. Members also suggested additional webinars beyond the 12-webinar training series, further underscoring the need for and importance of the I-STARR work. The webinars in the training series have indeed been popular, generally attracting between 30 and 100 live attendees with many more viewing training recordings. Live webinars for both tracks of the training series ended in May 2024, but the entire series is housed on the I-STARR website, where those wishing to register for it can complete it asynchronously through the I-STARR learning management system. To date, we have had six operators and one researcher attend/view all 12 webinars in the respective training tracks. Further, the project continues to host monthly webinars to address additional topics raised by Advisory Board members and to facilitate dialog between researchers and operators. We also use the I-STARR website to provide resources (e.g., an annotated bibliography of key recovery housing research, links to relevant tools and texts, and a calendar of upcoming and recurring learning and networking opportunities, etc.) to researchers and recovery housing operators beyond what may be provided during I-STARR webinars.

### 4.3 Limitations

When evaluating the impact of this work to develop a roadmap for maximizing the use and effectiveness of recovery housing for individuals prescribed MOUD, some limitations must be kept in mind. With respect to our approach, we blended traditional approaches and took liberties in our operationalization of both the CHNRI and the James Lind Alliance priority setting frameworks, mostly to expedite the priority setting processing and limit burden on our Advisory Board members. That said, this hybrid approach also capitalized on the strengths of both techniques. Reviews of priority setting activities have found that approaches like ours, those that combine participatory and researcher-driven elements as well as surveys and consensus building activities, are commonly used (58–60). Additionally, while the membership of the Advisory Board was purposely curated to enhance diversity in training, background, and expertise on these topics, only 17 individuals participated in this priority setting exercise (completing the survey and/or participating in discussion at the meeting). As such, research and training priorities may not reflect all individuals with an interest or stake in this area. Further, it is possible that topics discussed at the meeting were raised by those with particular concerns or issues as well as by those who may have felt more comfortable raising them at the time. Finally, the recovery housing landscape and issues confronted by operators and researchers is ever-evolving and this work represents a snapshot of concerns in time. These limitations notwithstanding, it is important to note that conversations between the I-STARR team and the Advisory Board, as well as between the team and the researcher and operator communities, are ongoing. Although the research and training needed to support it may evolve, this work to set priorities serves as a valuable starting point.

## 5 Conclusion

Recovery housing could be an important resource for those prescribed MOUD as part of their recovery, but research supporting the effectiveness of recovery housing for this population is lacking. Guided by the input of a diverse group of stakeholders serving as Advisory Board members, the I-STARR project identified key research and training priorities to advance science on recovery housing for those prescribed MOUD. Pilot studies are already addressing key gaps in the literature, and training (including ongoing monthly webinars) continues to equip the field to tackle ongoing challenges.

## Data Availability

The raw data supporting the conclusions of this article will be made available by the authors, without undue reservation.
